# Risk Association of *TOX3* and *MMP7* Gene Polymorphisms with Sporadic Breast Cancer in Mexican Women

**DOI:** 10.3390/curroncol29020086

**Published:** 2022-02-11

**Authors:** Orlando Solis-Coronado, Mónica Patricia Villarreal-Vela, Hazyadee Frecia Rodríguez-Gutiérrez, Juan Francisco González-Guerrero, Ricardo M. Cerda-Flores, Fernando Alcorta-Núñez, Karen Paola Camarillo-Cárdenas, Diana Cristina Pérez-Ibave, Oscar Vidal-Gutiérrez, Genaro A. Ramírez-Correa, María Lourdes Garza-Rodríguez

**Affiliations:** 1 Servicio de Oncología, Centro Universitario Contra el Cáncer (CUCC), Hospital Universitario “Dr. José Eleuterio González”, Universidad Autónoma de Nuevo León, Av. Francisco I. Madero y Av. Gonzalitos s/n, Mitras Centro, Monterrey 64460, Mexico; orlando.solisc@gmail.com (O.S.-C.); hazyadee@gmail.com (H.F.R.-G.); juanfglz@hotmail.com (J.F.G.-G.); ferchoalcorta9@gmail.com (F.A.-N.); dianics83@gmail.com (D.C.P.-I.); vidal_oscar@hotmail.com (O.V.-G.); 2 Facultad de Ciencias Biológicas, Universidad Autónoma de Nuevo León, Av. Pedro de Alba s/n, Ciudad Universitaria, San Nicolás de los Garza, Monterrey 66450, Mexico; monicavv2@gmail.com (M.P.V.-V.); karencamarillo01@gmail.com (K.P.C.-C.); 3 Facultad de Enfermería, Universidad Autónoma de Nuevo León, Av. Dr. José Eleuterio González 1500, Mitras Centro, Monterrey 64460, Mexico; ricardocerda_mx@yahoo.com.mx; 4 Department of Molecular Science, University of Texas Health Rio Grande Valley, McAllen, TX 78502, USA; genaro.ramirezcorrea@utrgv.edu; 5 Department of Pediatrics, Division of Cardiology, Johns Hopkins University School of Medicine, Baltimore, MD 21205, USA

**Keywords:** breast cancer, *TOX3*, *MMP7*, SNP, polymorphisms, association analysis, triple-negative breast cancer, progesterone receptors, metalloproteinases

## Abstract

Breast cancer (BC) has one of the highest incidences and mortality worldwide. Single nucleotide polymorphisms (SNPs) in *TOX3* rs3803662 and *MMP7* rs1943779 have been associated with susceptibility to BC. In this case-control study, we evaluated the association of rs3803662 (*TOX3*)/rs1943779 (*MMP7*) SNPs with clinical features, immunohistochemical reactivity, and risk association with BC in women from northeastern Mexico. We compared 212 BC cases and 212 controls. DNA was isolated from peripheral blood to perform the polymerase chain reaction-restriction fragment length polymorphism (PCR-RFLP) assay. We calculated genotype frequencies, odds ratios, and 95% confidence intervals. We found that CT (Cytocine–Thymine) and TT (Thymine –Thymine) genotypes, and T alleles of *TOX3* rs3803662, were associated with BC risk (*p* = 0.034, *p* = 0.011, respectively). SNP *TOX3* rs3803662 was associated with positive progesterone receptors (PR) and triple-negative BC (TNBC) but not with estrogen receptor (ER) or HER2 reactivity. CT and TT genotypes (*p* = 0.006) and T alleles (*p* = 0.002) of SNP *MMP7* rs1943779 were associated with risk of BC. We found that T alleles of *TOX3* rs3803662 and *MMP7* rs1943779 SNPs are associated with BC risk. These findings contribute to personalized medicine in Mexican women.

## 1. Introduction

Breast cancer (BC) is the second leading cause of death by neoplasia in women worldwide. In 2020, in the United States alone, 281,550 new cases and 62,470 deaths were registered. In recent years, the increased incidence and lethality rates have prioritized BC as a public health problem [1,2].

Current prevention strategies, such as breast self-examination and mammography, are still lagging behind the primary goal of early BC detection. Therefore, implementing highly sensitive and specific BC biomarkers is essential to combat the healthcare and economic burden [3,4].

BC is a multifactorial, heterogeneous, and complex disease. Multiple genetic, environmental, and socio-cultural risk factors interact together and contribute to the BC pathogenesis. Among these factors are age, ethnicity, socioeconomic status, reproductive, hormonal, and nutritional factors, and lifestyles that lead to overweight and obesity [5].

BC tumors are classified based on morphology, gene, and immunophenotypic expression, and are divided into four molecular groups: luminal A (express estrogen receptors [ER+], progesterone receptors [PR+], Human epidermal growth factor receptor 2 [HER2−]), luminal B (ER+, PR+ or HER2+/−), HER2+ (ER−, PR−, HER2+), and triple-negative BC (TNBC or basal-like) (ER−, PR−, HER2-). This classification predicts the behavior, aggressiveness, and response to treatment, and helps to understand tumor biology. Luminal subtypes A and B have the best prognosis, whereas HER2 and TNBC have the worst prognosis due to their high aggressiveness [6,7].

Sporadic BC represents 90% of the cases, whereas 10% have hereditary variants [1,8]. Genome-wide association studies (GWAS) have identified low penetrance polymorphic variants of a single nucleotide polymorphism (SNP) that increases the risk of BC [9]. For this reason, SNPs could be used as disease biomarkers and to achieve a better understanding of BC pathogenesis, early diagnosis, and personalized treatment.

For instance, SNPs in TOX High Mobility Group Box Family Member 3 (*TOX3)* (rs3803662) and Matrix Metallopeptidase 7 *(MMP7)* (rs1943779) have been linked to increased susceptibility of BC in females from different populations [10,11,12]. *TOX3* gene localizes on chromosome 16q12.1; it has seven exons and encodes the nuclear protein TOX3.

TOX3 induces transcription of estrogens and Bcl-2-sensitive promoters, and binds to a BRCA1 promoter region to downregulate its expression through methylation [13,14,15]. TOX3 expression is increased in BC tumor tissue when compared to healthy breast tissue, and its expression in progenitor cells of the mammary epithelium suggests an involvement in the initiation of BC. TOX3 participates in cell proliferation, migration, and survival after apoptotic stimuli [16,17].

The most common genetic variant of *TOX3* is the SNP rs3803662 (C > T), commonly linked to BC [14]. It has been reported that the T allele influences BC prognosis and is linked to advanced tumor stages, worse survival, and luminal molecular subtype or expressed ER+ [18,19].

The *MMP7* gene is a proto-oncogene involved in cell proliferation, tumor formation, and invasion [8]. The *MMP7* gene localizes on chromosome 11q22.2; it has six exons and encodes matrix metalloproteases family [20].

Metalloproteases are responsible for tissue remodeling and degradation of the extracellular matrix, thus explaining their importance in the pathogenesis of metastasis [21,22].

The present study analyzed the association between SNPs rs3803662 of the *TOX3* gene and rs1943779 of the *MMP7* gene with clinical-pathological variables of patients with BC in Mexico’s northeast region.

## 2. Materials and Methods

### 2.1. Study Design and Population

This work is a hospital case-control study approved by the Institutional Ethics Research Committee of the Hospital Universitario “Dr. José Eleuterio Gonzalez”- Universidad Autónoma de Nuevo León (UANL) in Monterrey, Mexico (protocol registration number BI10-002). The study was conducted under the principles of Helsinki’s statement.

We included 212 cases of women with histopathological diagnosis of BC (adenocarcinoma), and 212 healthy women with negative mammograms for BC as controls. All the patient’s parents and grandparents were born in Mexico. The cases were recruited at the Centro Universitario Contra el Cáncer (CUCC) of the Hospital Universitario “Dr. José Eleuterio González”—UANL. Controls were women older than 18 years old, without a history of cancer, and a BI-RADS 1–2 mammogram classification. All control group women were recruited in the radiology areas. They attended a follow-up mammography or were referred for early detection of BC by mammography. All participants signed an informed consent letter. Clinical and epidemiological data were obtained by interview and medical records. Peripheral blood samples for DNA extraction were taken of all participants.

Exclusion criteria for this study were: hereditary history of BC or another neoplasia, incomplete data from pathology or clinical reports, pregnant women, and comorbidities.

### 2.2. Primer Design and Restriction Enzymes Selection

We obtained a list of SNP sequences for *TOX3* and *MMP7* genes from the National Center for Biotechnology Information/Single Nucleotide Polymorphisms database (NCBI dbSNP) [23]. For this study, we chose the rs3803662 (NG_012623.1:g.374T>C) polymorphism of *TOX3* gene and the rs1943779 (NC_000011.9:g.102407191T>C) polymorphism of the *MMP7* gene. Primers were designed according to the nucleotide sequence published in the ENSEMBL database, and using OLIGO 7 software (Molecular Biology Insides, Inc., Cascade, CO, USA) [24]. The primer sequence is shown in Table 1.

The resulting sequences were analyzed using the NEBcutter V2.0 webpage to predict the restriction fragment length polymorphism (RFLP) banding patterns [25]. The main characteristics of the restriction enzymes, recognition sites, base pair fragments, and SNP localization are presented Table 1.

We performed the treatment with restriction enzymes *Bpu10I* for the *TOX3* rs3803662 and *HpyCH4IV* for SNP *MMP7* rs1943779 polymorphisms and observed the band patterns per lane in agarose gel electrophoresis, using a Gene Ruler DNA ladder (GeneRuler DNA Ladder Mix, Thermo Fisher Scientific Inc., Carlsbad, CA, USA). According to the digestion pattern of each enzyme and SNP, the individuals were classified into the following categories: homozygous CC, homozygous TT, and heterozygous CT.

### 2.3. Genomic DNA Isolation and Genotyping

Peripheral blood samples were collected in tubes with ethylenediaminetetraacetic acid (EDTA). Genomic DNA was purified from peripheral lymphocytes using the QIAmp DNA Blood Kit (Cat No. 51,104 Qiagen Inc., Santa Clarita, CA, USA) according to the manufacturer’s instructions. DNA concentration and quality were evaluated by NanoDrop 8000 (Thermo Fisher Scientific Inc., Wilmington, DE, USA). PCR-RFLP assay was used to determine the genotypes of *TOX3* rs3803662 and *MMP7* rs1943779 polymorphisms. PCR reaction was performed in a total volume of 25 μL containing: 100 ng of genomic DNA, 1 U GoTaq DNA Polymerase (Promega Corporation, Madison, WI, USA), 1x reaction buffer (750 mM Tris-HCl pH 9.0, 500 mM KCl, 200 mM NH_4_2SO_4_, 400 μM of each dNTP, 3 mM MgCl_2_, and 200 μM of forward and reverse of each primer. Forward and reverse primers for both polymorphisms were acquired from IDT (Integrated DNA Technologies Inc., Coralville, IA, USA).

PCR amplifications were conducted using the Veriti 96-well thermal cycler (Applied Biosystems, Foster, CA, USA). Thermal cycling conditions were as follows: initial denaturation step at 94 °C for 5 min, 35 cycles at 94 °C for 30 s, 58/60 °C for 30 s, and 72 °C for 60 s, and one cycle at 72 °C for 10 min for a final extension. The amplified products of the *TOX3* rs3803662 and *MMP7* rs1943779 polymorphisms were digested with 2.5 U of *Bpu10I* and 5 U of *HpyCH4IV*, respectively (New England Biolabs, Beverly, MA, USA). The PCR products were incubated at 37 °C for 12 h and then electrophoresed on a 1.5% agarose gel stained with ethidium bromide and viewed under a UV trans-illuminator.

### 2.4. Statistical Analysis

Statistical analysis was performed using the SPSS 27.0 statistical package (IBM Corporation). Distributions of genotypes and alleles between groups were tested using χ^2^ analysis. For each SNP, odds ratios (ORs), and 95 % confidence intervals (CIs) were calculated.

The Hardy–Weinberg equilibrium (HWE) was calculated with the χ^2^, using a public web page (http://dr-petrek.eu/links.html, accessed on 1 August 2021). HWE is a principle that states that genetic variation in a population will remain constant from one generation to the next in the absence of some evolutionary force. If there are factors that alter this balance, the genetic variation will be disturbed and the HWE will be *p* ≤ 0.05 (HW disequilibrium) [26].

## 3. Results

### 3.1. Clinical and Demographic Characteristics

We included 212 patients with a confirmed diagnosis of BC and 212 healthy controls. The case-control demographic characteristics are presented in Table 2. The mean age of cases and controls was 54.22 ± 12.06 and 52.10 ± 28.95 years old, respectively. There was no significant difference between the age of both groups. Obesity and alcohol consumption were more frequent in cases (*p* ≥ 0.0001, *p* = 0.025, respectively). Regarding menstrual status, 34.9% of BC patients and 62.7% of controls were premenopausal (*p* ≥ 0.001). We did not find statistically significant differences in other factors related to the development of BC, such as smoking, oral contraceptives, age of menarche, age of the first delivery, and hormone replacement therapy.

In terms of molecular characterization of BC, luminal A was the most predominant subtype (51.4%—109/212), followed by triple-negative BC (24.0%—51/212), luminal B-HER2 enriched BC (18.0%—38/212), HER2 (5.2%—12/212), and luminal B HER2-negative (1.0%—2/212). The presence of metastasis was reported in 21.2% (45/212) of the cases. Immunohistochemical reports are shown in Table 3.

### 3.2. PCR-RFLP Assays

**Figure 1:**
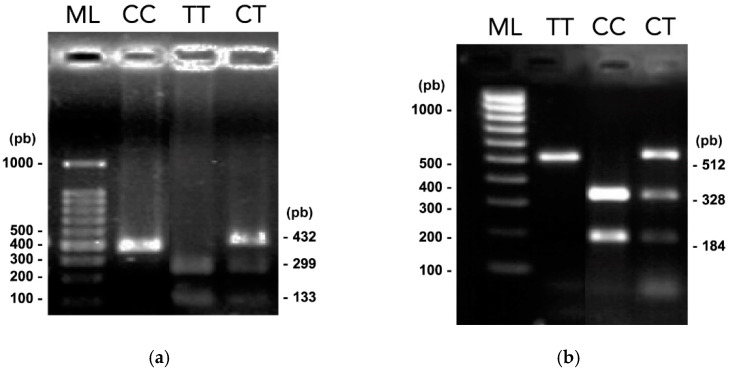
PCR-RFLPs results. Electrophoresis in 1.5% agarose gels of digested amplified products with *Bpu10I* and *HpyCH4IV* restriction enzymes (related to Figure S1) (**a**) Digested amplified products of SNP *TOX3* rs3803662. (**b**) Digested amplified products of SNP *MMP7* rs1943779. TT, CC, and CT genotypes are shown, and lane ML indicates the 100 to 1000 bp molecular gene ruler ladder.

For SNP *TOX3* rs3803662, band patterns that presented two DNA fragments (299 and 133 bp) were classified as homozygous TT. Homozygotes CC was observed as a 432 bp fragment, and those with three DNA fragments of 432, 299, and 133 bp, were heterozygotes CT. Furthermore, for SNP *MMP7* rs1943779, 328 and 184 bp DNA fragments were classified as homozygous CC; a fragment of 512 bp was homozygous TT, and finally, those with three DNA fragments of 512, 328, and 184 bp were heterozygous CT. Figure 1 represents the electrophoresis results of the PCR-RFLP assay.

### 3.3. Genotyping and Allelic Distributions in BC Cases and Controls

This study found a significant association between rs3803662 (*TOX3*) and rs1943779 (*MMP7*) SNPs with BC risk. Allelic and genotypic frequencies are presented in Table 4. Statistical analysis showed significant differences between cases and controls in their allelic and genotypic frequencies in both SNPs studied. For the variant rs3803662 (*TOX3*), the T allele was associated with an increased risk of BC (OR = 1.38, 95% CI = 1.054–1.813). Genotypic frequencies were statistically significant (*p* = 0.034). Furthermore, the heterozygous CT, and the homozygous TT of *TOX3* rs3803662, were associated with an increased risk of BC. Genotype frequencies for *TOX3* rs3803662 polymorphism were in equilibrium for the controls, according to the Hardy–Weinberg (HW) equation. In cases, *p*-value was <0.05, and was considered to be in HW disequilibrium (Table 4).

For the *MMP7* rs1943779 variant, the T allele was associated with an increased risk of BC (OR = 1.527, 95% CI = 1.138–2.824). Genotypic frequencies exhibited statistically significant differences (*p* = 0.006). CT and TT genotypes were associated with an increased risk of BC. Genotype frequencies for *MMP7* rs1943779 were in HW disequilibrium in controls (*p* ≤ 0.05). The cases were in HWE (Table 4).

Stratified analysis determined an association of *TOX3* rs3803662 polymorphism with PR+ expression (OR = 2436, 95% CI = 1063–5580) and with a higher risk of presenting TNBC (ER-/PR-/HER2-) (OR = 3884, 95% CI = 1317–11,456) in a dominant model (CC vs. CT/TT), but not with ER+ or HER2+ expression. We did not find an association of the *MMP7* rs1943779 variant with the ER, PR, and HER2 expression. There was no association between a dominant model (CC vs. TC/TT) with TNBC or metastasis in any analyzed SNPs (Table 5).

## 4. Discussion

BC is a complex health problem worldwide due to the increase in the number of new cases and deaths every year. One of the causes of this increase is the epidemic of obesity, the decrease in parity, changes in people’s social behavior, and lifestyles [1]. Despite the new treatments, improvements in diagnosis, and systematized preventive medicine programs, BC continues to be a challenge for health systems worldwide, mainly due to the high costs generated for its care. The search for new alternatives for detecting BC in early stages is valuable, highlighting the importance of studying cancer genetics [3]. In this regard, identifying allelic variants through SNPs may help to understand the biological mechanisms of development, invasion, and metastasis. The study of SNPs is helpful to make improvements in the prevention of BC and the implementation of personalized treatments to ameliorate fatality and incidence rates [4].

SNP studies have been based mainly on detecting genotypic variants through GWAS studies, especially in European and Caucasian populations. SNPs studies are important in Mexican people because the information is scare. Moreover, the Mexican population has a particular genetic admixture because of the mix of indigenous Mexican, African, and Spanish genomes in the population. These studies are relevant to understand the associations of complex pathologies, such as cancer, especially in genetically mixed populations, such as the Mexican population. Genomic studies allow us to analyze the impact on the susceptibility, or the protection from BC or other types of neoplasia in the Mexican mestizo population [9,27].

Our study found that the T allele of SNP *TOX3* rs3803662 is associated with a higher risk of developing BC in the mestizo northeastern Mexican population. Our finding coincides with those in Asian and Caucasian populations [28]. Similarly, meta-analysis and GWAS studies of various ethnicities, such as those in southwestern United States [29], Chile [27], Iran [14], Taiwan [30], Turkey [31], and Vietnam [10], demonstrated that the T allele of *TOX3* rs3803662 was associated with a higher risk for developing BC [11,15,32,33]. Contrary to our results, in studies in Afro-American [11,16,34,35], and Chinese and other Asian populations [12,15,36,37,38], the T allele of *TOX3* rs3803662 was not associated with BC risk. This discrepancy may be due to differences in the genetic architecture and allelic frequencies of different populations.

A similar study in the Asian population by He et al. [36] agreed with a study in a Mexican population by Figueroa et al., where no differences in the genotypes of the allelic variants and no association in the T allele of *TOX3* rs3803662 with BC susceptibility were found [39].

Figueroa et al. were the first to analyze the risk association of *TOX3* rs3803662 SNP in Mexican BC patients. This study included 56 cases and 83 controls from central Mexico. The study was in a region having a population with a different genetic mixture compared to the northeast. They found that *TOX3* rs3803662 polymorphism was not associated with BC. The contrast with our findings may be explained by the population size and the region of Mexico.

In addition, our study found an association between *TOX3* rs3803662 polymorphism and the PR expression and a higher risk of presenting a TNBC subtype. To identify if this polymorphism affects the development and biology of TNBC and PR+ patients, immunohistochemical and protein functionality studies are required. Interestingly, we did not find previous studies that describe the risk association between the variants rs3803662 of the *TOX3* gene and TNBC, which is an essential contribution of the present study. We also did not find an association between rs3803662, ERs, and HER2 expression. Other studies reported that this SNP is associated with ER expression and the development of bone metastasis [11,16,35,40].

Our data on *MMP7* rs1943779 SNP showed no risk association with the expression of cellular markers (ER, PR, HER2) or metastasis. On the contrary, AL-Eitan et al. found that *MMP7* rs1943779 SNP was associated with ER expression in a Jordan cohort [8], whereas another study reported that the *MMP7* SNP rs1943779 had a protective association against metastasis.

Our study reveals that the T allele of *MMP7* rs1943779 was associated with BC risk, coinciding with the findings reported in a cohort of more than 800 cases from the United Kingdom [41].

Notably, few studies in the world link the risk association of the SNP *MMP7* rs1943779 with BC. To our knowledge, the present work is the first to be carried out in a population from Latin America.

MMPs have a relevant function in the epithelial-mesenchymal transition, by degrading proteolytically the extracellular matrix [22,42]. It is essential to identify the biological mechanism by which MMP SNPs confer risk against BC.

To date, our study has the highest recruitment of cases analyzing both SNPs in Mexico. An essential advantage of this work is that we stratified our patients based on the presence of metastasis and the expression of ER, PR, or HER2, or the lack of expression in triple-negative cases. An additional advantage is that our population was grouped by age.

Lastly, this study makes it possible to identify two risk SNPs that could be potential risk biomarkers tools for BC, and may thus contribute to personalized medicine against BC.

## 5. Conclusions

The present study results indicate that the *TOX3* rs3803662 (C > T) polymorphism is associated with an increased risk of BC in northeastern Mexican women. Furthermore, this SNP is associated with PR expression and the TNBC subtype. We also report that the SNP *MMP7* rs1943779 (C > T) is associated with an increased risk of BC; however, we did not find an association with ER, PR, or HER2 expression, or with the TNBC subtype. In the stratified analysis, metastasis was not associated with any of the investigated SNPs. The information provided in this study can help to determine the risk profile of rs3803662 (*TOX3*)/rs1943779 (*MMP7*) polymorphisms and BC, and in this way contribute to personalized medicine.

## Figures and Tables

**Table 1:** SNP primers, genome localization, and restriction enzymes for RFLP assays.

Gene/SNP	Localization (NT Position) *	Alleles (WT/Var)	Primer Design		RE	Recognition site (5′- 3′) **	WT Amplicon
			NT Sequence	Amplicon (bp)			
*TOX3*/ rs3803662	Chr 16 (52552429)	C/T	F -5′ AGTCCTTGGCTGTTCTGTG 3′	465	*Bpu10I*	CCTNAGC	298,167
			R -5′ GTCCAGACAGTCTTCAGCAG 3′				
*MMP7*/ rs1943779	Chr 11 (102536460)	C/T	F -5′ CTGTGCTTCAAAAACACTGC 3′	514	*HpyCH4IV*	ACGT	328,184
			R -5′ TTTCTGTGGGTTGTCTTTCAC 3′				

* According to the Genome Reference Consortium Human Build 38 patch release 12 (GRCh38.p12). ** Nucleotide base in the underlined letter is the recognition site of the restriction endonuclease. NT: Nucleotide; Chr: Chromosome; WT: Wild-type; Var; Variant allele; F: Forward primer; R: Reverse primer; bp: base pairs; RE: Restriction enzyme.

**Table 2:** Demographic and clinical characteristics of cases and controls.

Characteristics	Cases *n* (%) *n* = 212	Controls *n* (%) *n* = 212	*p*-Value
Age (media ± SD)	54.22 ± 12.06	52.10 ± 28.95	0.327 *
Body Mass Index <30 >30			
	115 (54.2%)	155 (73.1%)	**>0.001**
	97 (45.8%)	57 (26.9%)	
Menarche (media ± SD)	12.72 ± 1.54 *	12.78 ± 1.45	0.708 *
Oral contraceptives Consumers Non-consumers			
	54 (25.5%)	52 (24.5%)	0.911
	158 (74.5%)	160 (75.5%)	
Hormonal Replace Therapy Consumers Non-consumers			
	18 (8.5%)	17 (8.0%)	0.862
	193 (91.5%)	195 (92.0%)	
Age at the first child (media ± SD)	22.13 ± 5.04	22.26 ± 5.03	0.796
Menopause			
Pre-menopause	74 (34.9%)	133 (62.7%)	**>0.001**
Post-menopause	138 (65.1%)	79 (37.3%)	
Smoking			
Smokers	34 (16.1%)	44 (21.3%)	0.209
Non-smokers	177 (83.9%)	163 (78.7%)	
Alcohol			
Yes	15 (7.1%)	29 (14.0%)	**0.025**
No	196 (92.9%)	178 (86.0%)	

Chi-square; * *t*-student; SD: Standard deviation.

**Table 3:** Distribution of immunohistochemical studies in BC patients.

IHC Status	ER (%)	PR (%)	HER2 (%)
Positive	147 (69.3%)	145 (68.4%)	53 (25.0%)
Negative	65 (30.7%)	67 (31.6%)	159 (75.0%)

IHC: Immunohistochemistry; ER: Estrogen receptor; PR: Progesterone receptor; HER2: Human epidermal growth factor receptor 2.

**Table 4:** Distribution of genotypes and alleles of *TOX3* and *MMP7* polymorphisms.

SNP	Genotype	Cases *n* (%) *n* = 212	Controls (%) *n* = 212	*p*-Value	OR	95% CI
*TOX3* rs3803662 (C > T)	CC	46 (21.7)	69 (32.5)	**0.034**	1	Reference
	CT	122 (57.5)	110 (51.9)		1.164	1.057–2.618
	TT	44 (20.8)	33 (15.6)		2.000	1.114–3.592
	*X* *^2^*	4.84	0.9961			
	*p*-value HWE	0.0278	0.3118			
	Allele					
	C	214 (50.5)	248 (58.5)	**0.011**	1	Reference
	T	210 (49.5)	176 (41.5)		1.38	1.054–1.813
*MMP7* rs1943779 (C > T)	CC	20 (9.4)	40 (18.8)	**0.006**	1	Reference
	CT	70 (33.0)	71 (33.5)		2.165	1.119–4.190
	TT	122 (57.5)	101 (47.6)		2.703	1.442–5.067
	*X* *^2^*	3.779	14.922			
	*p*-value HWE	0.0956	0.0003			
	Allele					
	C	110 (26)	151 (35.6)	**0.002**	1	Reference
	T	314 (74)	273 (64.3)		1.527	1.138–2.824

HWE: Hardy–Weinberg equilibrium, OR: Odds Ratio, CI: Confidence interval, *X^2^*: chi-square.

**Table 5:** Association of *TOX3* and *MMP7* polymorphisms with receptors’ expression and metastasis in BC cases.

IHC Status	Allele	*TOX3* rs3803662				*MMP7* rs1943779			
		CT/TT vs. CC				CT/TT vs. CC			
		Positive *n* (%)	Negative *n* (%)	OR	95% CI	Positive *n* (%)	Negative *n* (%)	OR	95% CI
ER	C	35 (79.5)	9 (20.5)	1		10 (58.8)	7 (41.2)	1	
	T	112 (66.7)	56 (33.3)	1.944	0.874–4.326	137 (70.3)	58 (29.7)	0.605	0.220–1.666
PR	C	36 (81.8)	8 (18.2)	**1**		9 (52.9)	8 (47.1)	1	
	T	109 (64.9)	59 (35.1)	**2.436**	**1.063**–**5.580**	136 (69.7)	59 (30.3)	0.488	0.180–1.327
HER2	C	8 (18.2)	36 (81.8)	1		4 (23.5)	13 (76.5)	1	
	T	45 (26.8)	123 (73.2)	0.607	0.263–1.405	49 (25.1)	146 (74.9)	0.917	0.286–2.943
TNBC	C	4 (9.09)	40 (90.9)	**1**		7 (41.17)	10 (58.8)	1	
	T	47 (27.97)	121 (72.02)	**3.884**	**1.317**–**11.456**	151 (77.43)	44 (22.5)	0.416	0.150–1.157
Metastasis	C	35 (79.5)	9 (20.4)	1		4 (23.5)	13 (76.4)	1	
	T	132 (78.5)	36 (21.4)	1.061	0.467–2.408	41 (21.02)	154 (78.9)	0.865	0.268–2.795

IHC: Immunohistochemistry; ER: Estrogen receptor; PR: Progesterone receptor; TNBC: Triple-negative breast cancer; OR: Odds Ratio; CI: Confidence interval.

## Data Availability

The data of each participant in this study, as well as the signed informed consent letter, can be obtained by contacting the corresponding author at the following email: maria.garzarg@uanl.edu.mx.

## Supplementary Materials

The following supporting information can be downloaded at: https://www.mdpi.com/article/10.3390/curroncol29020086/s1, Figure S1: Full image related to Figure 1.
