# Supplementary material for: Risk Association of TOX3 and MMP7 Gene Polymorphisms with Sporadic Breast Cancer in Mexican Women
Source: Curr Oncol. 2022 Feb 11;29(2):1008–17. doi: 10.3390/curroncol29020086 (PMC8870835; doi:10.3390/curroncol29020086)
Supplement: Supplementary file 1 [file curroncol-29-00086-s001.zip › curroncol-1434445-supplementary.pdf]

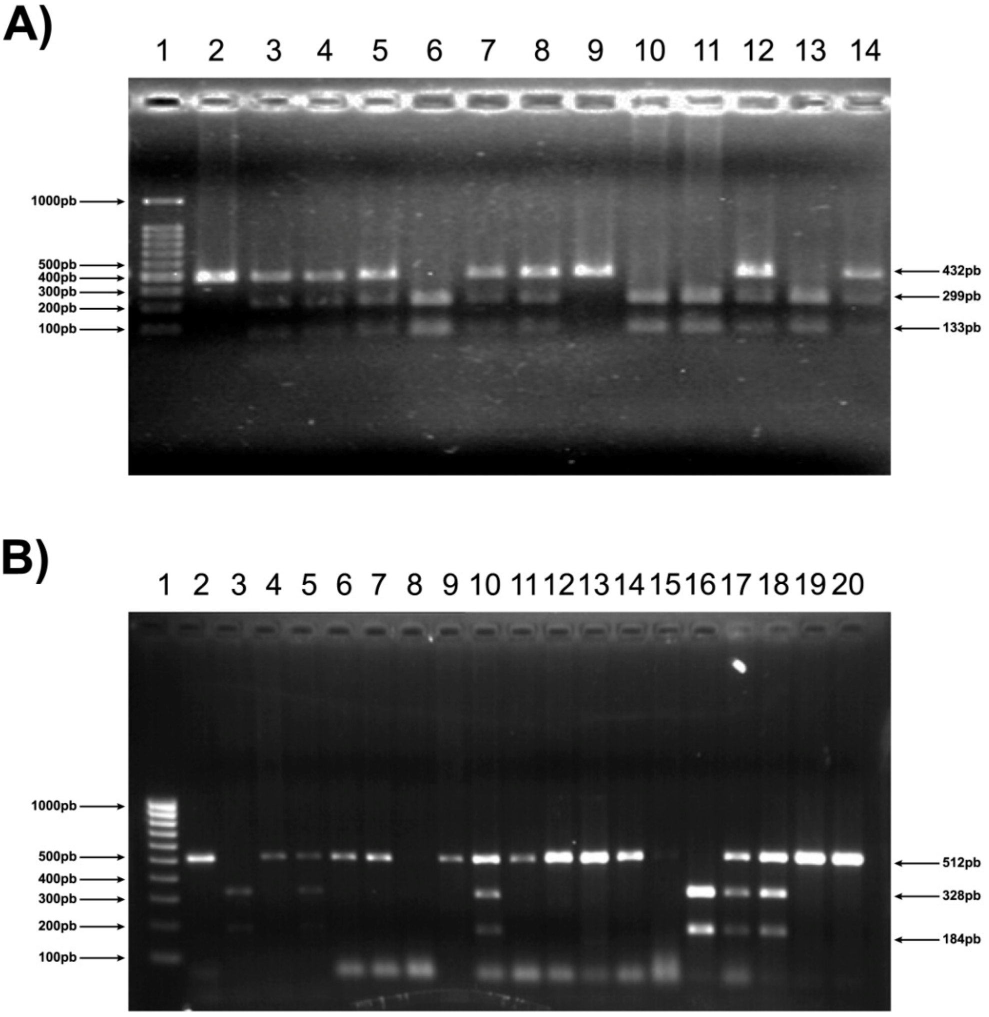

Figure S1. Full image related to Figure 1. PCR-RFLP amplified products of SNPs after digestion by the *Bpu10I* and *HpyCH4IV* restriction enzymes. A) Electrophoresis in agarose gel at 1.5% showing the amplified products for SNP *TOX3* rs3803662. Lanes 2 and 9 are genotyped CC; Lanes 6, 10, 11, and 13 are genotyped as TT Lane 3, 4, 5, 7, 8, 12, and 14 are genotyped as CT. B) Electrophoresis in agarose gel at 1.5% showing the amplified products for SNP *MMP7* rs1943779. Lane 3 and 16 is genotyped as CC; Lanes 2, 4, 6, 7, 9, 11, 12, 13, 14, 19, and 20 are genotyped as TT; Lanes 5, 10, 17, and 18 are genotyped as CT; Lane 1 in both cases indicates the 100 to 1000 bp molecular gene ruler.
